# Yams (*Dioscorea* spp.) in shellmounds and swiddens: ancient history in Babitonga Bay, Santa Catarina State, southern Brazil

**DOI:** 10.1186/s13002-024-00653-4

**Published:** 2024-02-02

**Authors:** Dalzemira Anselmo da Silva Souza, Dione Rocha da Bandeira, Nivaldo Peroni

**Affiliations:** 1 Graduate Program in Biology of Fungi, Algae and Plants, Universidade Federal de Santa Catarina, Florianópolis, Brazil; 2 Museu Arqueológico de Sambaqui de Joinville, Joinville, Santa Catarina Brazil; 3 Laboratory of Human Ecology and Ethnobotany, Universidade Federal de Santa Catarina, Florianópolis, Brazil; 4 Graduate Program in Cultural Heritage and Society, Universidade da Região de Joinville, Joinville, Santa Catarina Brazil; 5 Graduate Program in Ecology, Universidade Federal de Santa Catarina, Florianópolis, Brazil

**Keywords:** Shellmounds, Swiddens, Cultural niche, Domestication, Historical ecology

## Abstract

**Background:**

In Babitonga Bay, southern Brazil, records of yam consumption exist among shellmound builders from at least 4000 years ago. Shellmounds (s*ambaquis*) are anthropogenic structures in the form of mounds with layers of shells associated with other faunal remains, as well as with charcoal, artefacts and burial. Larger s*ambaquis* are considered to be funerary monuments. The indigenous *Jê* and *Guarani* people also lived in the region before the European invasion and cultivated yams. Currently, exotic and domesticated yams are cultivated in the region by farmers. Our aim is to describe the long-term history between the people and *Dioscorea* in the Babitonga Bay region based on its consumption and occurrence in shellmounds and swiddens.

**Methods:**

Surveys of *Dioscorea* spp. and host were carried out in the vegetation of shellmounds and in the surrounding area using visual detection through intensive searches in transects using the walking method. The survey of *Dioscorea* species used and cultivated in the precolonial, colonial and current periods was carried out based on the literature. In the present study, only *Dioscorea trifida* cultivations were recorded.

**Results:**

*Dioscorea cayennensis, Dioscorea chondrocarpa*, *Dioscorea dodecaneura*, *Dioscorea laxiflora*, *Dioscorea olfersiana,* and *Dioscorea scabra,* all recorded in associated vegetation of shellmounds, in different combinations of the species. In swiddens, *D. trifida* is most common, followed by *Dioscorea alata* and, to a lesser extent, *Dioscorea bulbifera* and *D. cayennensis*. Records of food use prevail, but they are used as medicinal plants. Yams are integrated on anthropogenic soils of shellmounds and in swiddens in monoculture systems or in intercropping with *Zea mays* or *Colocasia esculenta*. The presence of exotic food trees and *D. cayennensis* in some shellmounds indicates the influence of colonizers on the composition of the vegetation. In *sambaquis,* there are overlapping processes of construction of cultural niches by different human groups at different times.

**Conclusions:**

The *sambaquis* and the associated vegetation and swiddens form part of a domesticated landscape. The native species of *Dioscorea* recorded in shellmounds and surrounding vegetation do not depend on human action to perpetuate themselves in the environment. However, this does not rule out human influence in the past, but it does not indicate horticulture among the *Sambaquianos*. Greater investment in genetic, archaeobotanical and ethnobotanical research can contribute to a better understanding of the relationship between people and yams over thousands of years.

## Background

The formation of contemporary and past landscapes can be understood through interactions over time between societies and their environments [1], whose transformation can be explained through niche construction theory [2, 3].

Interactions between people and forests have occurred for thousands of years and can be perceived through patches of useful plants near archaeological sites [4–14]. In the southern Atlantic Forest, there is evidence of this interaction and the influence of different peoples and their cultures [15–21].

Shellmound builders (*Sambaquianos*) were fishermen [22] who lived and used plants from the ecosystems of the Atlantic Forest Biome [19, 23] between the middle and late Holocene [24]. Shellmounds (s*ambaquis*) are anthropogenic structures [25, 26] in the form of mounds with layers of shells associated with other faunal remains, as well as with charcoal, artefacts and burial [25]. Larger s*ambaquis* are considered to be funerary monuments [24], where feasts were held on the occasion of human burials [27]. Shellmounds represent important records for understanding human influence on the coastal landscape of Brazil [28]. Studying them provides an understanding of settlement patterns, the functions of sites, subsistence and social organization [25]. The archaeological plant remains in shellmounds provide important indications of diet [29]. Fragments of carbonized yam tubers from 6000 years BP (before present) have been recovered in the southeastern region [23]. In the southern region, *Dioscorea* starch grains in the *Morro do Ouro* shellmound in Babitonga Bay date to 4030 ± 40 years BP [30, 31] and those in Jabuticabeira shellmound date to 2880 ± 75–1805 ± 65 years BP, located in a lagoon complex [32, 33].

Yams are among humans’ oldest foods [34] and have been recorded in archaeological sites since the Pleistocene [35]. These foods rich in carbohydrates and sources of bioactive substances for medicinal use [36–38] are important in tropical regions [39].

The domestication of *Dioscorea* likely started with forest management through the selection of yam plants and the protection and gathering of tubers [34]. In tropical forests where perennial yam tubers are common, they provide a source of food for long periods [36, 40].

Of the more than 640 existing species of *Dioscorea* (Dioscoreaceae) [41], twelve are widely cultivated today, with an emphasis on African and Asian species [38, 42]. The use of *Dioscorea* tubers in Brazil is still minor, with a predominance of exotic *Dioscorea alata* L. and *D. cayennensis* Lam., both introduced in the sixteenth century by Portuguese colonizers, and of *Dioscorea bulbifera* L., introduced by Dutch settlers [43]. The Amazonian species *D. trifida* L.f., domesticated by indigenous people before European invasion, is currently cultivated mainly in northern, southern and southeastern Brazil [44, 45]. The consumption of native and wild yams in Brazil is linked to indigenous peoples and farmers, who use and cultivate them on a small scale [46, 47]. In Santa Catarina state, southern Brazil, the main yam-producing farmlands are located in the Babitonga Bay region [48]. The tradition is currently maintained by descendants of European immigrants, who in the nineteenth century incorporated yam cultivation from their contact with colonizers and indigenous people [49, 50].

Considering the ancient consumption of yam by *Sambaquianos* and the records of their intense and long-term interaction with the forest, are there species of *Dioscorea* among the vegetation today associated with these places that could have been consumed by them? How does the current consumption and cultivation of yam in the Babitonga Bay region relate to its ancient use by the native indigenous peoples who lived there, including the *Sambaquianos*? Thus, the objective of this study is to analyse the interactions of this long-term history between people and yams in Babitonga Bay and find current species of *Dioscorea* associated with different landscape units, especially concentrated in shellmounds and swiddens.

## Methods

### Study area

**Fig. 1:**
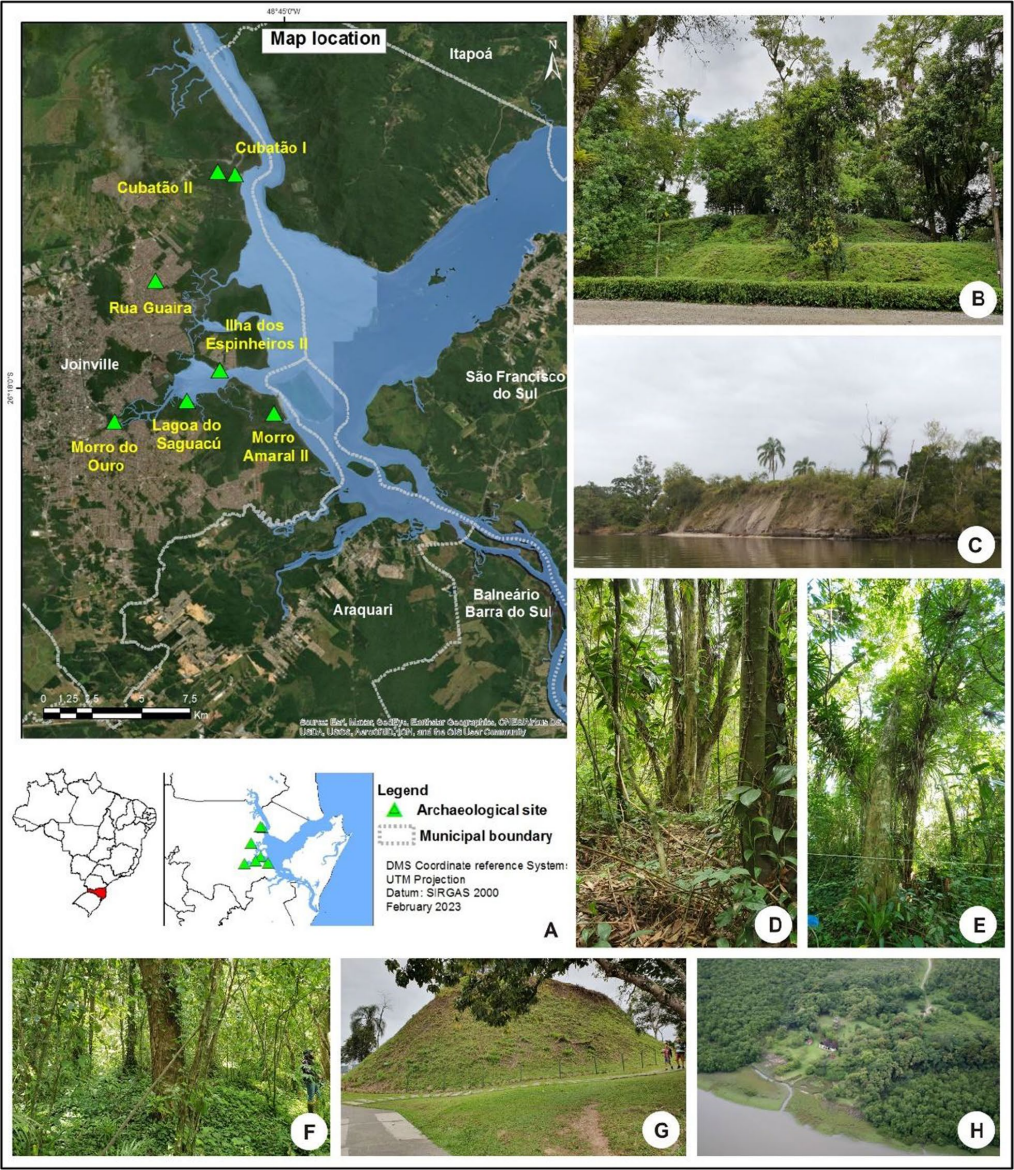
Shellmounds in Babitonga Bay, Joinville, Santa Catarina state, selected for the study. **A** Map with location of the shellmounds around Babitonga Bay; **B** *Ilha dos Espinheiros* II; **C** *Cubatão I*; **D** *Rua Guaíra*; **E** *Cubatão II*; **F** *Morro do Amaral* II; **G** *Morro do Ouro*; **H** *Lagoa Saguaçu*

The study area is located in the municipality of Joinville, located on the northern coast of Santa Catarina state, southern Brazil (Fig. 1). The ecosystems in this region are part of the Atlantic Forest Biome, with a predominance of mangroves, Restinga forest, and Dense Ombrophilous Forest (FOD) distributed around Babitonga Bay [51]. In Joinville, 41 shellmounds are registered [52], most of which are found around this estuary [53] (Fig. 1). According to the classification proposed by Köppen, Joinville is classified as having a mesothermal climate, as the temperatures in the coldest month are below 18 °C and above 3 °C and classified as humid, as it does not have a defined dry season [51].

Seven shellmounds were selected for the present study (Fig. 1; Table 1) based on the following criteria: location in the region of Babitonga Bay; presence of forest component in their surroundings; history of interventions in its structure and vegetation composition, such as the exploitation of shells for the production of lime and the carrying out of archaeological excavations. In addition, *Morro do Ouro* shellmound was included due to the identification of archaeobotanical material that attested to the consumption of *Dioscorea* sp. [31].

**Table 1:** Description of the shellmounds surveyed in the present study, Joinville–Santa Catarina state. *Source*: adapted from Joinville [52]

Shellmound	Location and History
*Cubatão*** I** Dating: 3480 ± 80 years BP [19] Dimensions: 130 × 90 × 9.0 26° 12′ 17″ S 48° 46′ 20″ W	Located at the mouth of the Cubatão River in Babitonga Bay. Shellmound is constantly waterlogged and contains preserved archaeological plant artefacts. Archaeological excavations occurred in 2007, 2009 and 2020
*Cubatão*** II** Dating: no date** Dimensions: 60 × 70 × 1.5 26° 12′ 11″ S 48° 46′ 49″ W	Located five meters from the *Cubatão* III shellmound and less than 1 km from *Cubatão* I. Lost part of its structure due to road construction
*Ilha dos Espinheiros*** II** Dating: 3,015 ± 130 years BP [53] Dimensions: 80 × 40 × 5 26° 17′ 31″ S 48° 46′ 45″ W	Located on the shores of Babitonga Bay. Archaeological excavation occurred in the 1980s. The owner periodically removes the forest understory
*Lagoa Saguaçu* Dating: 4690 ± 30 years BP [60] Dimensions: 130 × 180 × 9 26° 18′ 20″ S 48° 47′ 39″ W	Located on the shores of Babitonga Bay, much of its structure was removed due to the exploitation of shells for the manufacture of lime until the mid-twentieth century
*Morro do Amaral*** II** Dating: 2998–2756 years cal BP*** Dimensions: 20 × 70 × 3.5 26° 18′ 38″ S 48° 45′ 16″ W	Located on Morro do Amaral Island on the shores of Babitonga Bay. There is no record of significant changes in its structure. On the island, there is a community of artisanal fishermen, descendants of Luso-Brazilian settlers in the seventeenth century
*Morro do Ouro* Dating: 4030 ± 40 years BP [61] Dimensions: 60 × 95 × 13 26° 18′ 53″ S 48° 49′ 40″ W	Located at the mouth of the Cachoeira River in Babitonga Bay. It was exploited for lime production until the mid-twentieth century. Part of the structure was removed to build a road. Archaeological excavations occurred in 1952–1960, 1968, 1979 and 2019
*Rua Guaíra* Dating: 5200 years ± 70 years BP [52] Dimensions: 40 × 40 × 16 26° 15′ 07″ S 48° 48′ 32″ W	Located on the slope of a small hill in a densely occupied and urbanized region, approximately 3 km from Babitonga Bay. There is a historical record of material removal from its structure

*Greatest width (meters) × length (meters) × highest point (meters) according to surveys carried out by Oliveira [53]

**Not dated, but it is associated with the *Cubatão* III shellmound, dating to 3930 ± 60 years BP (Before the Present)

***Dated in present study

### *Dioscorea* surveys

The surveys of *Dioscorea* spp. were carried out in the vegetation above the shellmounds and in the surrounding area. On the shellmounds, individuals of *Dioscorea* spp. and host were registered by walking [54] and wide patrolling methods [55]. The vegetation in the shellmounds and surrounding areas was characterized based on the recording of tree species obtained using the walking method. Around the *Cubatão* I shellmound, the survey was stratified into two subareas, where CUB I-A is the portion with forest and CUB I-B is the portion covered by grasses and sparse tree vegetation. In the vegetation surrounding the shellmounds three transects, 20 m apart, are with up to 500 m in length from the edge of the site. Up to a distance of 5 m on both sides of each transect, visual detection of *Dioscorea* was performed [56] through intensive search by two or three observers [57]. In the *sambaquis Morro do Amaral* II, *Rua Guaíra* and *Cubatão* I, the surrounding forest is contiguous to their edges, while in the others, it starts at different distances from these structures. In the *Lagoa Saguaçu* shellmound, the forest starts beyond 100 m of a wide mangrove strip; in *Morro do Ouro* shellmound at 30 m beyond the road; in *Ilha dos Espinheiros* II at 20 m beyond a parking lot; and in *Cubatão* II beyond a 10-m-wide stream. We did not search in the forest around the s*ambaqui Cubatão* I.

The survey of *Dioscorea* species used in the precolonial, colonial, and current periods was carried out with Google Scholar. The keywords used were *precolonial history of Joinville and São Francisco do Sul; Dioscorea or yam in Santa Catarina; colonization of Joinville and São Francisco do Sul; and indigenous ethnobotany in Santa Catarina*. Additionally, the research relied on literature borrowed from local historians.

The survey of *Dioscorea* species cultivated in the study area was based on data from previous research in the region by Santos [48], Veasey et al. [44], Siqueira [58] and Nascimento [59], who identified *D. trifida* as the main species cultivated.

In this research, we only recorded the cultivation of *D. trifida* in the rural area of Joinville, which is divided into two main regions: Pirabeiraba and Piraí, which have 8 and 14 roads, respectively, that provide access to rural properties. To record the cultivation of *D. trifida*, 10 km was covered in 2020 on three roads in the Piraí region and 51.3 km on 10 roads in the Pirabeiraba region. Fields with *D. trifida* were recorded along the roads.

The nomenclature of *Dioscorea* species and other plant species was according to Angiosperm phylogeny classification of flowering plants (APG IV). The botanical collections in this study have been deposited in the FLOR Herbarium at the Federal University of Santa Catarina. The vouchers are available for online consultation at: http://flor.jbrj.gov.br/v2/consulta.php or Specieslink.net/search/. You can search for vouchers in this database using the filters: *Dioscorea*; municipality of Joinville; collector Souza, D.A.S. (first author of this article).

## Results

### Ancient consumption and cultivation of *Dioscorea*

The first record of *Dioscorea* consumption in the study area is 4030 ± 40 years BP by *Sambaquianos*, based on the identification of starch grains extracted from dental calculi in human remains recovered from *Morro do Ouro* shellmound [31]. From the post-*Sambaqui* culture period (1000 years BP) to the period of contact with colonizers in the sixteenth century, there is little information in the literature about the plant resources used by the indigenous peoples of *Jê* and *Guarani* in Babitonga Bay. According to the literature, these two indigenous groups consumed *Dioscorea* tubers and cultivated plants in other regions of the state of Santa Catarina [62, 63].

The oldest record of European contact in this region is by Binot Palmier de Goneville in 1504 [64]. According to the account of this French explorer, he found the *Guarani*, then called *Carijós*, with dozens of villages where they cultivated cassava (*Manihot esculenta* Crantz) and other roots and tubers, probably sweet potato, *Ipomoea batatas* **(**L) Lam., and yams (*Dioscorea* spp.). The use of *Dioscorea* in Babitonga Bay appears in historical records only from the nineteenth century onwards, when there is mention of the cultivation of *D. alata* by Luso-Brazilian colonizers, when the naturalist Saint Hilaire visited *Vila de São Francisco* (currently São Francisco do Sul municipality) in 1820 [65]. Another record is the cultivation of *D. bulbifera* reported by Theodor Rodowicz-Oswiecimsky in 1851 in the newly formed *Colônia Dona Francisca* [50], which today is the Joinville municipality. According to this visitor, the German colonizers learned to cultivate yams with the Luso-Brazilians and their slaves.

### Current cultivation of *Dioscorea*

The species of yam cultivated in the region of Babitonga Bay for local farmers, according to Santos [48], Veasey et al. [44], Siqueira [58] and Nascimento [59], are *D. alata, D. bulbifera, D. cayennensis* and *D. trifida* (Table 2). The main cultivated species are *D. alata* and *D. trifida* [48]. Air-yam (*D. bulbifera*) and *cará-de-espinho* ou Guinea-yam (*D. cayennensis*) are cultivated on a smaller scale, according to research by Santos [48].

**Table 2:** *Dioscorea* spp. in swiddens. Joinville–Santa Catarina state

Species	Folk name	Roads	Farm land*
*D. alata*	*cará-pão*	Dona Francisca^a,c^, Quiriri^c^	3
*D. bulbifera*	*cará-moela*	Quiriri^a,b^	1
*D. cayennensis*	*cará-de-espinho*	Dona Francisca^b^, Quiriri^b^, Pico^b^	3
*D. trifida*	*cará-mimoso*	Cubatão Grande^e^, Dona Francisca^a,d,e^, Guilherme^e^, Isaac^e^; João Fleith^e^, Morros^e^, Oeste^b^, Pico^b^, Quiriri^a,b^, Rio da Prata^b,e^	14

^a^Santos [48]; ^b^Veasey et al. [44]; ^c^Siqueira [58]; ^d^Nascimento [59]; ^e^Recorded in the present study

*Number of farmlands registered. One swidden per farmland was registered

In this study, we recorded the cultivation of *D. trifida* in seven farmlands, one on *Estrada dos Morros*, in the Piraí region, and six in the Pirabeiraba region (Table 2). *Dioscorea trifida* is cultivated in swiddens, where it is wrapped around stakes planted by farmers. As observed by Santos [48], it is also intercropped with *Zea mays* L. (corn), which serves as a host, and intercropping is also performed with *Colocasia esculenta* (L.) Schott. (*taro*).

### Occurrence of *Dioscorea* in the vegetation of shellmounds

**Fig. 2:**
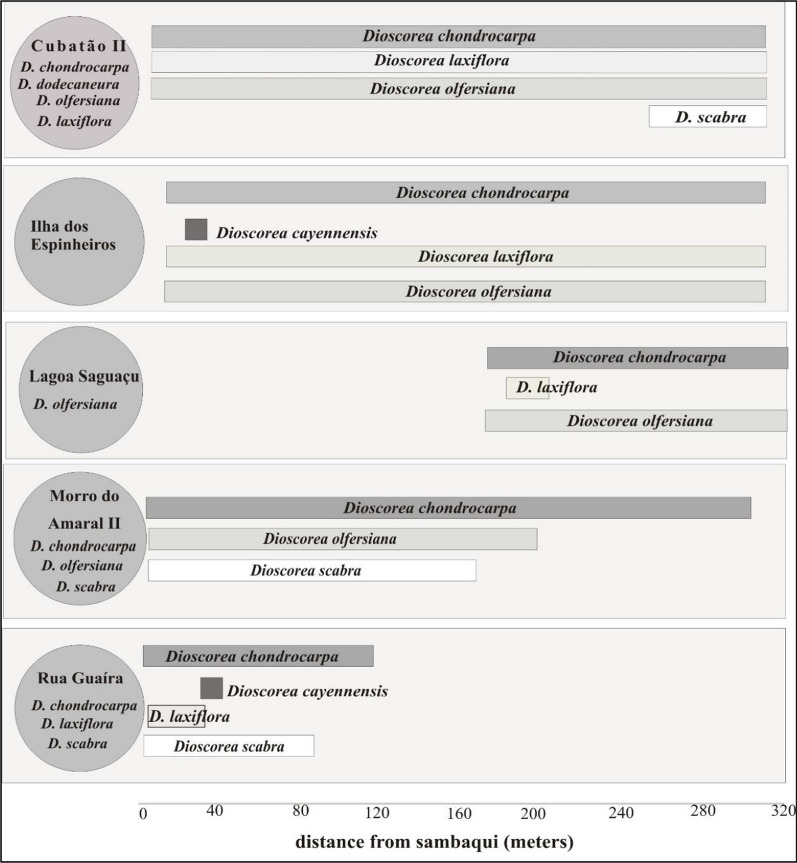
Graph showing the distribution of *Dioscorea* species on the shellmounds and along the transects in the surrounding vegetation

**Fig. 3:**
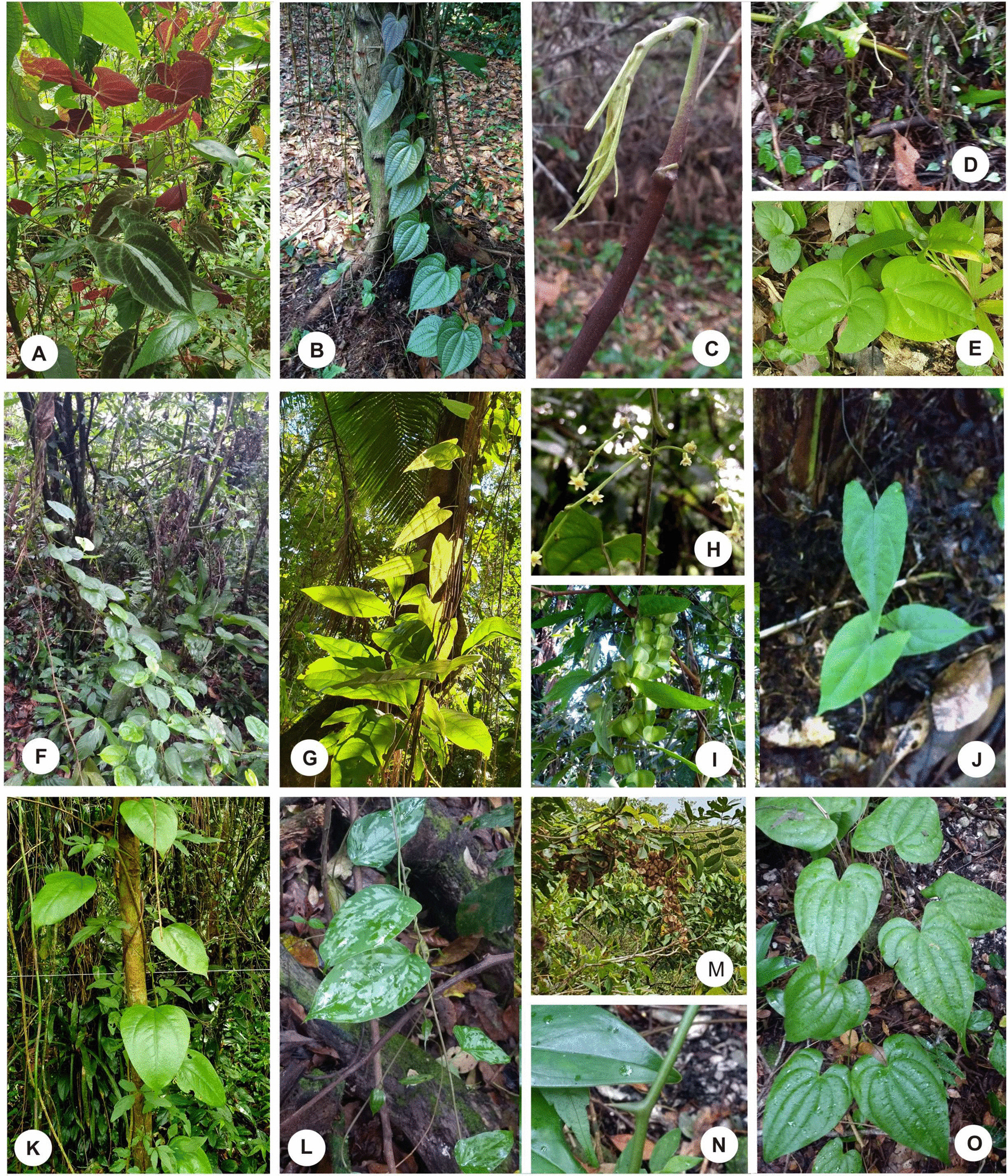
*Dioscorea* species associated with the shellmounds surveyed, Joinville–Santa Catarina state. **A** *D dodecaneura* in *Cubatão* I; **B** *D. chondrocarpa* in the vegetation around *Ilha dos Espinheiros* II; **C** apical portion of the secondary stem of *D. chondrocarpa* recorded in the vegetation around *Morro do Amaral* II; **D** seedling bank of *D. chondrocarpa* recorded in the vegetation around *Lagoa Saguaçu*; **E** seedling of *D. olfersiana* on *Morro do Amaral* II*;* **F** *D. cayennensis* in the vegetation around Rua *Guaíra*; **G** *D. laxiflora* on *Cubatão* II: **H** *D. laxiflora* with fruits on *Cubatão* I; **I** *D. laxiflora* with flowers; **J** seedling of *D. laxiflora* in the vegetation around *Ilha dos Espinheiros* II; **K** *D. olfersiana* on *Cubatão* II; **L** *D. olfersiana* in the vegetation around *Ilha dos Espinheiros* II; **M** *D. olfersiana* with fruits in *Cubatão* I-A; **N** detail of the stem thorn of *D. scabra* on *Morro do Amaral* II; **O** *D. scabra* between the shells of *Morro do Amaral* II shellmound

From April 2019 to August 2021, six species of *Dioscorea* were recorded in the *sambaquis* and surrounding forests in different combinations at each site. Five are native, *Dioscorea chondrocarpa, Dioscorea dodecaneura, Dioscorea laxiflora, Dioscorea olfersiana and Dioscorea scabra,* and the exotic *D. cayennensis* (Table 3; Figs. 2, 3).

**Table 3:** *Dioscorea* species recorded in the vegetation on the shellmounds and surroundings in the study area

Species	Folk name	Shellmounds and surrounding area													
		CUB I-A	CUB I-B	CUB II	ECUB II	ILES II	EILES II	LAS	ELAS	MA II	EMA II	MO	EMO	RGU	ERGU
Native															
* D. chondrocarpa*	*cará-de-espinho*	0	0	1	1	0	1	0	1	1	1	0	0	1	1
* D. dodecaneura*	*caratinga-roxa*	1	0	1	0	0	0	0	0	0	0	0	0	0	0
* D. laxiflora*	*caratinga-brava*	1	0	1	1	0	1	0	1	0	0	0	0	1	1
* D. olfersiana*	*cará*	1	0	1	1	0	1	1	1	1	1	0	0	0	0
* D. scabra*	*cará*	0	0	0	1	0	0	0	0	1	1	0	0	1	1
Exotic															
* D. cayennensis*	*cará-de-espinho*	0	1	0	0	0	1	0	0	0	0	0	0	0	1

*CUB I-A* portion of *Cubatão* I shellmound with forest, *CUB I-B* portion of *Cubatão* I shellmound with grasses and sparse tree vegetation. *CUB II Cubatão* II shellmound, *ECUBII Cubatão* II surrounding area, *ILES II Ilha dos Espinheiros* II shellmound, *EILES II Ilha dos Espinheiros* II surrounding area, *LAS Lagoa Saguaçu* shellmound, *ELAS Lagoa Saguaçu* surrounding area, *MA II Morro do Amaral* II shellmound, *EMAII Morro do Amaral* II surrounding area, *MO Morro do Ouro* shellmound, *RGU Rua Guaíra* shellmound, *ERGU Rua Guaíra* surrounding area.1: this *Dioscorea* sp. Present; 0: this *Dioscorea* sp. Absent

All shellmounds are surrounded by Restinga Forest, with the exception of *Rua Guaíra*, where there is a Dense Ombrophilous Forest (FOD). This shellmound and surrounding areas, the following tree species were identified, which according to Flora e Funga do Brasil [66] are FOD indicator species: *Hieronyma alchorneoides* Allemão (*licurana*)*, Nectandra membranacea* (Sw.) Griseb. (*canela-branca*), *Hirtella hebeclada* Moric. ex DC. (*cinzeiro*) and *Endlicheria paniculata* (Spreng.) J.F. Macbr*.* (*canela-frade*). In the other shellmounds, tree species were recorded that, according to Falkenberg [67], are important elements of the Restinga Forest: *Alchornea triplinervia* (Spreng.) Müll. Arg. (*tanheiro*), *Andira fraxinifolia* Benth. (*angelim*), *Calophyllum brasiliense* Cambess. (*olandi*), *Eugenia astringens* Cambess. (*jabuticaba-da-praia*), *Nectandra oppositifolia* Nees (*canela-ferrugem*), *Ocotea pulchella* (Nees & Mart.) Mez. (*canelinha*), *Psidium cattleyanum* Sabine (a*raçá*), *Tapirira guianensis* Aubl. (*copiúva*), *Syagrus romanzoffiana* (Cham.) Glassman (*jerivá*), and others.

In the shellmounds with greater intervention in their structure and vegetation from European colonization to the present, such as the exploitation of shells for the production of lime and the carrying out of archaeological excavations, *Cubatão* I–B, *Ilha dos Espinheiros* II, *Lagoa Saguaçu* and *Morro do Ouro*, we recorded several exotic food trees among Atlantic Rainforest species (Table 4).

**Table 4:** Exotic food trees registered on the shellmounds discussed in this study

Family/Exotic tree	Folk name	CUB I-A	CUB I-B	CUB II	ILES II	LAS	MA II	MO	RGU
Ebenaceae									
* Diospyros nigra* (J.F.Gmel.) Perrier	*sapota-preta*	0	0	0	1	0	0	0	0
Moraceae									
* Morus nigra* L	*amora-preta*	0	0	0	0	0	0	1	0
Lauraceae									
* Persea americana* Mill	*abacate*	0	1	1	1	1		1	0
Myrtaceae									
* Psidium guajava* L	*goiabeira*	0	1	0	1	1	0	1	0
* Syzygium cumini* (L.) Skeels	*jambolão*	0	0	0	1	0	0	1	0
* Syzygium jambos* (L.) Alston	*jambo-amarelo*	0	0	0	0	0	1	0	0
Rosaceae									
* Eriobotrya japonica* (Thunb.) Lindl	*nespereira*	0	0	0	1	1	0	0	0
Rutaceae									
* Citrus x limon* (L.) Osbeck	*limão*	0	0	1	0	1	1	0	0

*CUB I-A* portion of *Cubatão* I with forest, *CUB I-B* portion of *Cubatão* I with grasses and sparse trees, *CUB II Cubatão* II, *ILES II Ilha dos Espinheiros* II, *LAS Lagoa Saguaçu*, *MA II Morro do Amaral* II, *MO Morro do Ouro*, *RGU Rua Guaíra*. 1: this tree sp. present; 0: this tree sp. absent

In the vegetation associated with shellmounds, we recorded that the *Dioscorea* species wrap themselves around different hosts, the most frequent being *Bactris setosa* Mart. (*tucum*), *Cyathea phalerata* Mart. (*samambaiaçu*), *Davilla rugosa* Poir. (*cipó-caboclo*), *Geonoma schottiana* Mart. (*guaricana*), *Guatteria australis* A.St.-Hil. (*cortiça*), *Monstera adansoni* Schott. (*costela-de-adão*), *Philodendron appendiculatum* Nadruz & Mayo (*cipó-imbé*), and *Tapirira guianensis* Aubl. (*copiúva*).

**Fig. 4:**
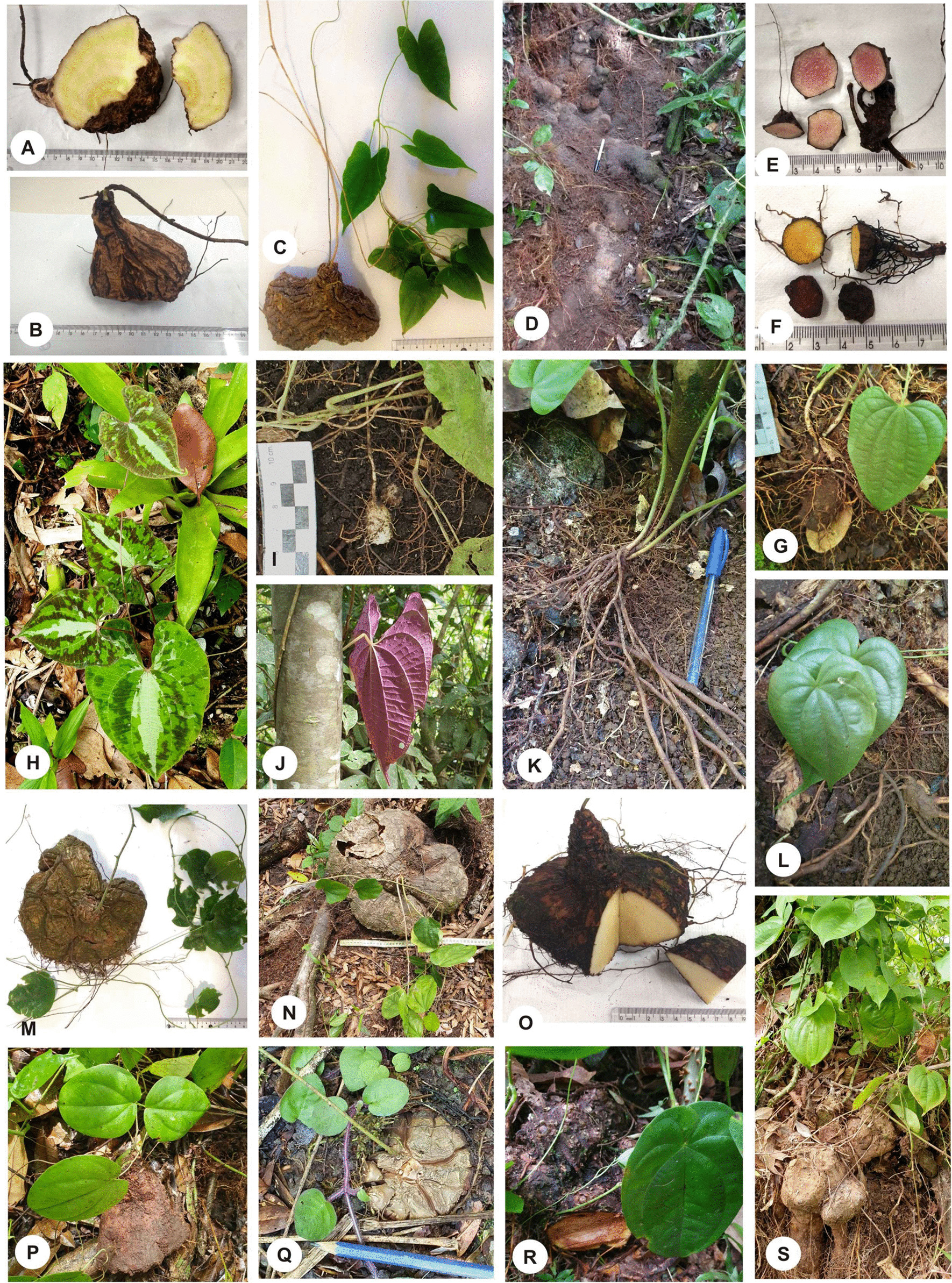
Tuberized subterranean organs of *Dioscorea* species associated with the surveyed shellmounds, Joinville–Santa Catarina state. **A**–**C** tubers of *D. laxiflora* collected in the surrounding vegetation of *Ilha dos Espinheiros* II; **D**–**F** tubers of *D. chondrocarpa* recorded in the surrounding vegetation of *Ilha dos Espinheiros* II; **G** young tuber of *D. chondrocarpa* in the vegetation around *Morro do Amaral* II*;* **H**, **J** detail of variegated leaves and purple abaxial face of *D. dodecaneura* on *Cubatão* I-A; **I** tuber of *D. dodecaneura* on *Cubatão* II; **K, L** rhizophores of *D. scabra* in the vegetation around *Rua Guaíra*; **M**–**P** tubers of *D. olfersiana* recorded in the vegetation around *Ilha dos Espinheiros* II; **Q** tubers of *D. olfersiana* recorded in the vegetation around *Cubatão* II; **R**, **S** tubers of *D. cayennensis* recorded in the vegetation around *Ilha dos Espinheiros* II and *Cubatão* I-B, respectively

We recorded yam species with aerial stems in both the warmer and colder seasons of the year. Sexual reproduction in *Dioscorea* species was recorded from the presence of seedlings and, less frequently, flowers and fruits, which were observed only in *D. laxiflora* and *D. olfersiana* (Fig. 3). The native *D. chondrocarpa, D. laxiflora, and D. olfersiana* have perennial tubers and *D. scabra* perennial rhizophores [47]. *D. dodecaneura* renews its tubers annually [68]. Among cultivated species in the region, only *D. cayennensis and D. bulbifera* have perennial tubers [44]. In *D. laxiflora,* tubers are pyriform, the largest measuring approximately 8 cm in the widest part and with yellow pulp (Fig. 4A–C). In *D. chondrocarpa*, the tubers are globose to oval, measuring up to 5 cm in diameter, with yellow or purple colour, connected to a woody tuberous system (Fig. 4D–F). In *D. dodecaneura,* tubers are globose, measuring approximately 4 cm in diameter and with white pulp (Fig. 4I). *D. scabra* has elongated, fibrous, and thin rhizophores (Fig. 4K, L). In *D. olfersiana,* the tubers present a discoid or piriform shape and have a yellowish-white pulp. They were observed with varying sizes, between 12 and 43 cm in the widest portion (Fig. 4M–Q). In the species *D. cayennensis*, branched tubers with yellow colour and larger than those of native species were recorded (Fig. 4R, S).

### Uses of *Dioscorea* species

Records of food use prevail in the literature, but there is also information on medicinal use for *Dioscorea* species that we recorded in wild conditions and on farms [37, 43, 44, 46–48]. *Dioscorea trifida* was identified as the most used species within the study area, followed by *D. alata* in the research by Santos [48]. The air yam (*D. bulbifera*) and the *cará-de-espinho* or Guinea-yam (*D. cayennensis)* are rarely used according to the research by Santos [48], a situation that remains in the studies by Veasey et al. [44], Siqueira [58] and Nascimento [59]. According Santos [48], *D. trifida* is consumed cooked and has the tastiest tubers according to farmers. The plant is also used as a healing agent [48]. According to Chu and Figueiredo-Ribeiro [46], *D. trifida* is used for digestive problems and asthma. The species *D. bulbifera* and *D. alata* are consumed cooked [48] and used to treat skin conditions and as diuretics due to their medicinal properties [46]. In Babitonga Bay, they are used in the preparation of bread [48]. Tubers of *D. cayennensis* are consumed cooked and have medicinal uses [44]. According to Barroso et al. [69], the tubers of *D. dodecaneura* can be eaten raw and have the flavour of almonds. In addition to being consumed boiled or roasted, *D. dodecaneura* is used in the production of an indigenous fermented drink called *chicha* [47] and in the treatment of skin conditions, rheumatism, diabetes and as a cardiotonic [46]. Among the wild species registered, only *D. laxiflora* is considered toxic according to Chu and Figueiredo-Ribeiro [46]. This species is used for female fertility and skin infections [46]. Tubers of *D. laxiflora, D. olfersiana* and *D. scabra* contain diosgenin [46], a precursor of female hormones and are widely used in the pharmaceutical industry [36]. *Dioscorea scabra* has compounds with antibacterial and antifungal activity [37].

## Discussion

### *An ancient story between Dioscorea* spp.* and people in Babitonga Bay*

**Fig. 5:**
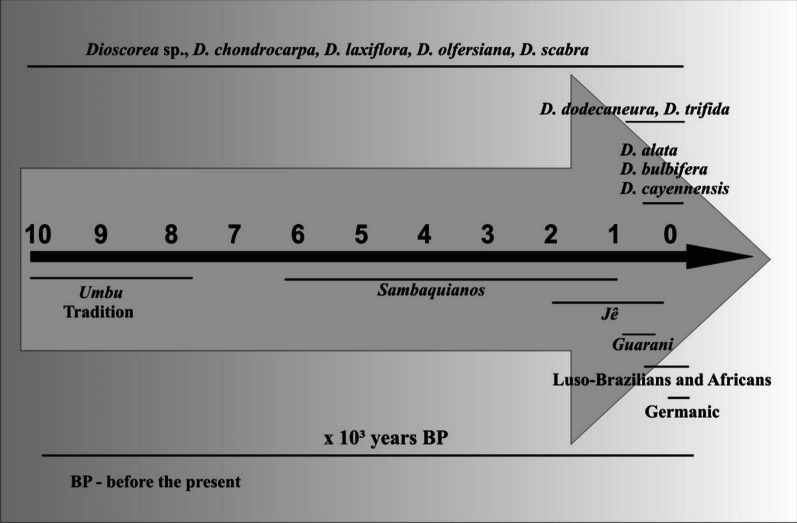
Timeline with recorded *Dioscorea* species in the study area and precolonial and colonial human groups

Yams are among humans’ oldest foods [34] and have been recorded in archaeological sites since the Pleistocene [35]. Archaeological remains of *Dioscorea* have been recovered from sites in Brazil dating back to the early Holocene [70], a period that in Babitonga Bay is related to the presence of hunter-gatherers from the *Umbu* tradition dating from 11,000 to 8000 years cal BP [71, 72] (Fig. 5).

The history between yams and people in Babitonga Bay began thousands of years ago, and even today, we find plants producing these tubers associated with places built at different times by different people and their cultures. The oldest record of *Dioscorea* consumption in Babitonga Bay is among the *Sambaquianos* in 4030 ± 40 years BP [31]. Another ancient record of yam consumption among *Sambaquianos* was found in the south of Santa Catarina [33]. In southeastern Brazil, charred archaeological fragments of *Dioscorea* tubers were found in shellmounds dating back to approximately six thousand years BP [23].

The shellmounds we researched date back to between 5200 and 2900 years BP (Table 1). Dating indicates that the construction of s*ambaquis* in Babitonga Bay occurred between 6 thousand and 1000 years BP [21, 73]. Radiocarbon dating indicates the presence of indigenous people *Jê* in Babitonga Bay from 1500 years BP [74]. The *Guarani* left their records in archaeological sites in Babitonga Bay from 400 years BP [74] (Fig. 5), a period in which European colonizers were already present in the region [64, 75, 76]. There are archaeological and ethnoarchaeological records that the *Jê* and *Guarani* indigenous groups also consumed *Dioscorea* in other regions of the state of Santa Catarina [62, 63, 77]*.* The oldest record of European contact in Babitonga Bay is by Binot Palmier de Goneville in 1504 [64]. According to the account of this French explorer, he found the *Guarani,* then called *Carijós*, with dozens of villages where they cultivated cassava (*Manihot esculenta* Crantz) and other roots and tubers, probably sweet potato, *Ipomoea batatas* **(**L) Lam., and yams (*Dioscorea* spp).

**Fig. 6:**
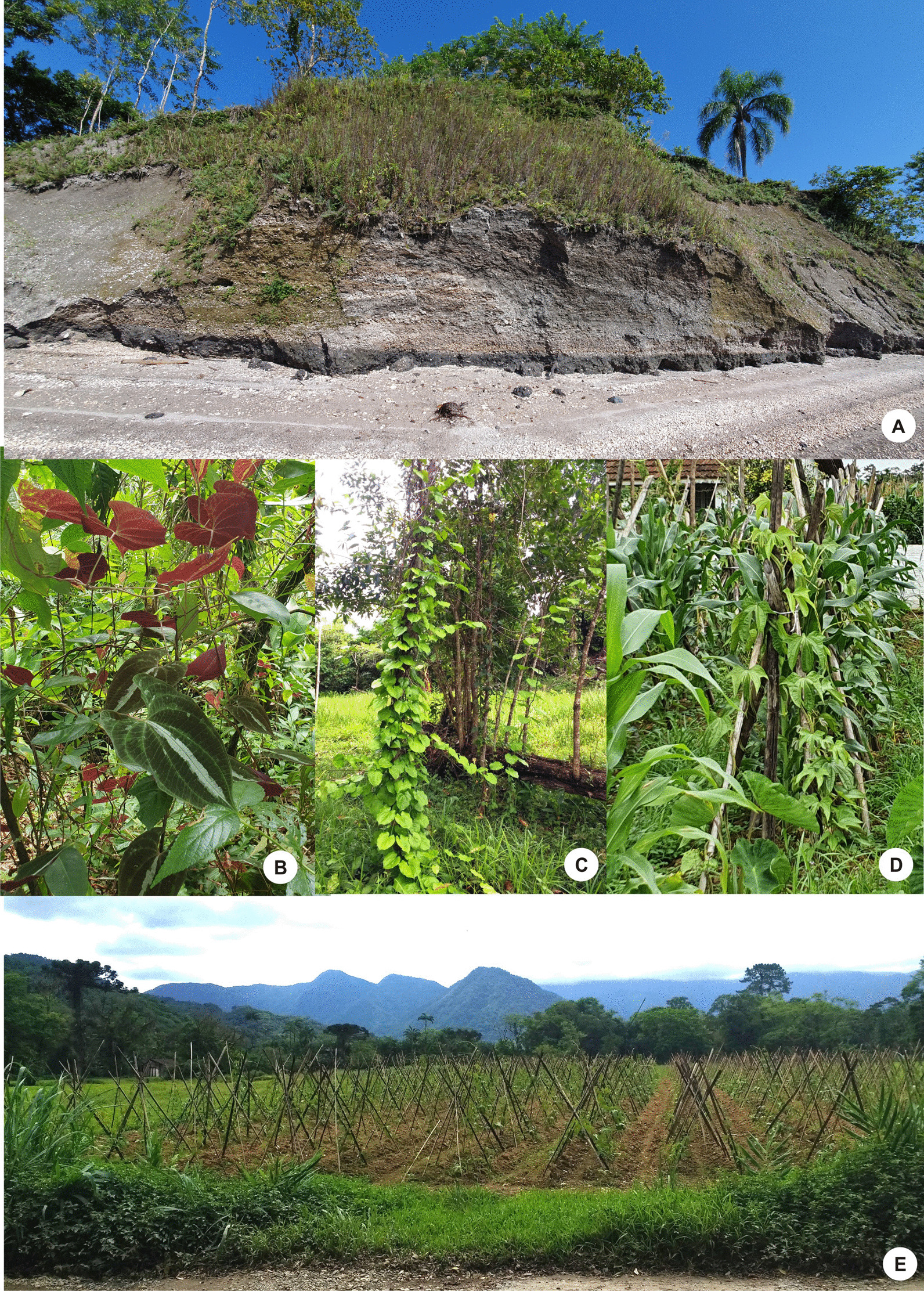
*Dioscorea* species in shellmounds and swiddens. **A** Shellmound *Cubatão* I—B; **B** *Dioscorea dodecaneura* over *Cubatão* I—A; **C** *D. cayennensis* over *Cubatão* I—B; **D** *D. trifida* in swiddens on Cubatão Grande Road; **E** *D. trifida* in swiddens on João Fleith Road

We recorded species of *Dioscorea* associated with *sambaquis* and swiddens in the Babitonga Bay region that maintain different relationships with people (Figs. 5, 6). Associated with the shellmounds are wild species of *Dioscorea*—*D. chondrocarpa, D. dodecaneura, D. laxiflora, D. olfersiana* and *D. scabra*—and the exotic *D. cayennensis*. In the swiddens, domesticated yams, *D. alata* (Asian), *D. cayennensis* (African), *D. bulbifera* (Asian and *African*) and *D. trifida* (Amazonian) [44, 48, 58, 59].

*Dioscorea trifida* and *D. dodecaneura* are among the yam species cultivated by *Guarani* [62, 77]. These and other *Dioscorea* species are associated with the *Guarani* plant pack, a set of plants and their propagules (seeds, roots, tubers and others) carried by them in their expansion to new territories, including southern Brazil, 2000 years BP [62]. *Dioscorea trifida* is from the Amazon rainforest [78] and was domesticated by indigenous people before the European invasion. Genetic studies indicate that accessions of *D. trifida* from Babitonga Bay are linked to the Amazon and that their origin is related to *Guarani* expansion [59] (Nascimento 2013). In the Amazon region, according to Clement [78], *D. dodecaneura* is considered semidomesticated and a food source for indigenous peoples before European invasion, in addition to domesticated *D. trifida*.

*Dioscorea chondrocarpa, D. dodecaneura, D. laxiflora, D. olfersiana* and *D. trifida* began to be cultivated by farmers in Brazil through contact with indigenous peoples [46, 47]. By 1550, the African yam (probably *D. cayennensis*) was already cultivated in southeastern Brazil by colonizers and their slaves, but the indigenous people preferred the native yams that they already cultivated and because they had better flavour [79]. This may also have been the scenario in Babitonga Bay.

The exotic species *D. cayennensis*, *D. alata* and *D. bulbifera* arrived in Brazil with colonizers from the sixteenth century onwards [43]. In Babitonga Bay, the historical record of cultivation of *D. alata* [65] and *D. bulbifera* [50] was initiated by Luso-Brazilians who arrived in the region in the seventeenth century [49, 76, 80]. Currently, they are cultivated by descendants of the Germanic colonizers who arrived in the region in the nineteenth century [44, 48, 58, 59] (Fig. 5).

The lack of historical records on the consumption of yams native to the region by colonizers does not mean that it did not occur but may be related to the type of use that Luso-Brazilian and Germanic colonizers made of the forest in the nineteenth century. According to Saint Hilaire [65] and Rodowicz-Oświęcimsky [50], in the region, food came from swiddens with domesticated species, such as the abovementioned species, and forests were intensively cut down to build villages. At the same time, some shellmounds were dismantled to extract the shells, which lasted until the mid-twentieth century [52]. These two actions resulted in the loss of vegetation and native *Dioscorea* species and the introduction of exotic species, as observed in some shellmounds (Table 3, 4 and Fig. 2).

Despite the strong association of native species of *Dioscorea* with shellmounds and diverse uses, there is no evidence or current records in the literature that the descendants of the colonizers have appropriated these species. Currently, descendants cultivate domesticated yams, and forest areas are replaced by different crops. Wild yam is a plant with great potential for use and is apparently not known. An important aspect to be considered in possible future uses of the vegetation associated with shellmounds is that they are archaeological sites protected by law, and interventions are prohibited without prior authorization by the National Institute of National Historical and Artistic Heritage (IPHAN, Brazil).

Considering that the exotic African and Asian species of yam arrived in Brazil only after colonization in the sixteenth century, we can infer that the *Sambaquianos, Jê* and *Guarani* consumed the native species of *Dioscorea*, and among these, the native species that currently occur are associated with shellmounds and *D. trifida* is associated with swiddens.

### Yams in a domesticated landscape

The Babitonga Bay landscape is home to approximately 170 *sambaquis* [73] that are integrated into the Restinga forests and mangroves. Among the s*ambaquis* researched, the exception is the *Rua Guaíra*, which was built on the slope of a small hill and is currently associated with the Dense Ombrophilous Forest. However, although they are currently integrated into the abovementioned ecosystems, they contain plant species from other regions of Brazil and even other countries. For example, in some shellmounds, we recorded exotic food plants related to European colonization (Table 4). In the case of *Persea americana* (avocado) and *Psidium guajava* (guava), which are not native to the region, they were probably already cultivated in Brazil before colonization [81, 82]. In the vegetation on and around the *sambaquis,* we recorded six species of *Dioscorea*, five native and the exotic *D. cayennensis*, African yam. The occurrence of plants of different origins indicates human influence at different times that have moulded the current landscape.

The expressive presence of *sambaquis* in the current landscape refers to an intense interaction between their builders and the environment. Archaeobotanical studies in shellmounds indicate that *Sambaquianos* mainly uses Restinga plants [23], including in Babitonga Bay [19]. However, Archaeobotany is a recent area of research in Brazil [83], and this is reflected in the small collection of archaeological food plant remains identified in shellmounds. Most of the archaeobotanical remains identified in *sambaquis* are from tree species from charcoal [19, 23, 84]. The first use mentioned is as fuel, but it is clear that many of the identified species produce edible fruits. The Myrtaceae family, which is very diverse in Restinga and well represented in archaeological remains [23], produces fruits that are highly appreciated by people. Examples are species of the genera *Psidium* and *Eugenia* [23], which we recorded to be associated with the *sambaquis* studied as *Psidium cattleyanum* (*araçá*) and *Eugenia astringens* Cambess (*jabuticaba-da-praia*). We recorded other plant species from Restinga associated with shellmounds, previously identified archaeological remains in Babitonga Bay. Trees such as *Andira fraxinifolia* and palm *Syagrus romanzoffiana* were identified by Melo Junior et al. [19] and Oliveira and Melo Junior [84]. Of the remains identified as Araceae by Wesolowski et al. [31] and *Philodendron* [85], we recorded *Monstera adansoni* and *Philodendron appendiculatum* in the current vegetation. Food plants such as *Dioscorea* spp. also identified by Wesolowski et al. [31] we registered in shellmounds *D. chondrocarpa*, *D. dodecaneura*, *D. laxiflora* and *D. olfersiana*, and medicinal plants *D. scabra*. All of these plants and countless others, with different uses for people, are in wild conditions and do not depend on human action to reproduce, persist and perpetuate in the environment.

Direct identification of plant consumption among *Sambaquianos* was carried out through the extraction of remains in dental calculi by Wesolowski [30] and Boyadjian [32] and by stable isotopes Pezo-Lanfranco et al. [86] and Toso et al. [87]. Among the remains, starch granules of *Dioscorea* sp. were identified, others suggestive and similar to *Ipomoea batatas* (sweet potato), *Zea mays* (corn) and *Araucaria angustifolia* [31, 33]. According to these authors, other remains, including starch granules and phytoliths, were identified as Marantaceae, Araceae and Poaceae.

Given the archaeobotanical research cited above, there is no doubt that the *Sambaquianos* used and consumed plants and that the consumption of carbohydrates was significant, as noted by Wesolowski [30] and Pezo-Lanfranco et al. [86]. Based on this reduced archaeobotanical data set of evidence for plant consumption, Pezo-Lafranco et al. [86] in their research and in review articles Scheel-Ybert and Boyadjian [29] and Scheel-Ybert et al. [88] suggest a mixed economy among *Sambaquianos* with the cultivation of domesticated plants.

Although there has been a record of plant cultivation in Brazil since 12 thousand years BP [82], it is still premature to confirm horticulture and the cultivation of domesticated plants among shellmound builders. Even with the spread of horticulture and agriculture, indigenous groups choose not to do so. In the Neotropics, there are several systems for obtaining food plants that are independent of cultivation and domestication [82]. In the lowlands of South America, of the 6261 plant species used, only 0.7% are completely domesticated, and gathering represents 98.6% of the way to obtain useful plants, among which 53% are trees [82]. Edible tubers of *Dioscorea* species can be collected and managed in the forest and cultivated [36, 40, 44, 46, 47, 89]. For example, the indigenous Nukak people in the Amazon form wild orchards where they promote some species over others, thus increasing the productivity of the forest [90].

There are wild species of *Dioscorea* [43, 46, 47], *Ipomoea* [91, 92] and Poaceae [93] that are used in food and that could simply be gathered and managed. It is not always easy to distinguish between remains, such as starch grains and phytoliths, from wild and domesticated plants [93, 94].

Currently, native species of *Dioscorea* recorded in shellmounds and surrounding vegetation perform sexual reproduction and do not depend on human action for their perpetuation in the environment. However, this does not rule out human influence in the past. Interactions between people and forests have occurred for thousands of years and can be perceived through patches of useful plants near archaeological sites [4–14]. The current vegetation that we have recorded in the shellmounds in Babitonga Bay developed on mounds built by humans. *Sambaquis* are anthropogenic structures [25] and intentionally built [24], with high fertility similar to that of Amazonian Dark Earth (*Terra Preta de Índio*) in the Amazon [26] and may harbour vegetation different from that found in nonanthropic soils.

In s*ambaquis* and swiddens, we record species of *Dioscorea* that are food and medicinal, according to the literature [37, 43, 44, 46–48]. The yams of shellmounds, *Dioscorea scabra,* a medicinal plant [37], is endemic to Brazil [95] and the most frequent *Dioscorea* in the state [47]. *Dioscorea olfersiana* is endemic to the Atlantic Forest, with sparse occurrence in Santa Catarina state [95] and cultivated in other regions of Brazil, as are *D. chondrocarpa* and *D. laxiflora* [46, 47]. Santa Catarina state appears to be the southern limit in the Atlantic Forest for *D. chondrocarpa* and *D. olfersiana* [95].

The five native species of *Dioscorea* that we recorded in the vegetation associated with shellmounds (Figs. 2, 3, 4; Table 3) are on the coastal plain, a few meters above sea level and associated with the Restinga Forest and Dense Lowland Rainforest. However, according to Pedralli [47] and Couto and Fraga [95], these native species have been recorded preferably in the hillside forests of the Atlantic Forest. In addition to being outside the preferred environment, we registered different combinations of *Dioscorea* among shellmounds in the same type of phytophysiognomy. This species composition may be related to the preference and manipulation of human groups in the past. A scenario similar to that proposed by Cruz et al. [20] when analysing the current vegetation of the Atlantic Forest in the state of Santa Catarina close to archaeological sites suggests a process of cultural niche construction.

The species *D. dodecaneura* before our research had only four records in SpeciesLink [96] and only one in Reflora [97] for the state of Santa Catarina. According to Pedralli [47], it is a rare species in Santa Catarina state and is possibly threatened with extinction, but in other regions of Brazil, it is cultivated. Considering its rarity, its preference for hillside forests in other regions of this biome [96, 97], and its occurrence restricted to the *Cubatão* I and *Cubatão* II *sambaquis* may indicate mediation by the *Guarani* indigenous peoples thousands of years ago in their expansion of Amazonia, as indicated by Noelli [62] and Pereira et al. [77]. According to Oliveira et al. [98], *D. dodecaneura* occurs in the vegetation associated with the archaeological site they studied in the southeastern region of Brazil and is currently used by the local community.

We recorded tubers of *D. dodecaneura* and *D. laxiflora* with sizes similar to those described for these species in wild conditions according to Barroso et al. [69], that is, they come from seeds that weigh a maximum of 200 g. In cultivation, through vegetative propagation, tubers of both species can reach up to 2.0 kg [47]. According to Onwueme [99], plants originating from tuber fragments have faster and more vigorous growth than those originating from seeds. Cultivation promotes an increase in yams through nutritional enhancement and better light conditions [36, 38] but also through the selection of phenotypes of interest to humans [9, 14]. The vegetative propagation of small fragments left in food preparation areas is one way to increase the density of *Dioscorea* plants in a location [40]. Shellmounds as funerary monuments built over thousands of years [24], where large parties were held during the burial of the dead [27]. The recurrent preparation and consumption of yams near these sites could have formed a concentration of these plants by vegetative reproduction. Charred fragments of *Dioscorea* tubers were recovered from shellmounds in southeastern Brazil by Scheel-Ybert [23].

An interesting characteristic that we observed among the *Dioscorea* species that we recorded in shellmounds is the perenniality of the tubers. The perennial nature of *Dioscorea* tubers is common in tropical forests [36]. The perenniality of the tubers associated with the formation of *Dioscorea* patches guarantees a prolonged source of food for local people, as reported by Yasuoka [40] and Scarcelli et al. [89] in Africa, by Head et al. [100], Atchison and Head [101, 102] in Australia, and Ferreira et al. [103] in Brazil. *Dioscorea* patches are gardens in the territory, ancestral places that are not routinely visited, they are in the memory of people, whose practices involving the care, gathering and replanting of tubers transform the landscape [100–102]. Among the six species we recorded associated with shellmounds and forests, five have perennial tubers or rhizophores. The exception is *D. dodecaneura*, which renews its tubers annually and in a similar way to *D. trifida*, according to Couto et al. [68]. In this sense, it can be proposed that the *Sambaquianos* and the other people who succeeded them were able to manage yams in the forests, which corroborates the statement by Toso et al. [87] that the *Sambaquianos* consumed plants harvested from the local forests. However, considering that in the forests of the region, we have recorded wild species that are currently cultivated in other regions of Brazil, the cultivation of yams among the *Sambaquianos* cannot be ruled out.

Among the native species associated with shellmounds, only *D. laxiflora* is cited in the literature as toxic [46, 104]. *D. laxiflora* has steroidal sapogenins, most of which are diosgenin [46, 69]. According to these authors, together with other secondary compounds such as toxic alkaloids, sapogenins act in the defence against herbivory. At least the majority of the toxins in *Dioscorea* appear to belong to one of three main categories: alkaloids, tannins and saponins [36]. For Corrêa [43] and Chu and Figueiredo-Ribeiro [46], *D. laxiflora* is edible, but not cultivated, is gathered and managed. However, for Pedralli [47], *D. laxiflora* is cultivated and used by small farmers in the state of Minas Gerais, Brazil. According to Corrêa [43], the tuber of *D. laxiflora*, known as *caratinga-brava* or *cará-de-sapo*, needs to go through a long cooking process to become edible. According to Coursey [36] and Lebot [38], there are traditional ways to remove toxicity from tubers before consumption, such as immersion in water and ash for a few days. The toxicity of a plant does not prevent its use. *D. laxiflora* flour of the cooked tuber is mixed with corn flour to produce bread [43]. The folk name also indicates possible toxicity, similar to what is recorded by Peroni et al. [105] in *Manihot esculenta* (cassava), where the term bitter or *brava* (*mandioca-brava*) in Portuguese refers to the variety that has the highest concentration of toxin. However, in addition to steroidal sapogenins, *D. laxiflora* may have other toxic secondary compounds, and therefore, further studies are necessary.

The tubers of many species of *Dioscorea* are highly toxic and may be used deliberately as sources of poison for hunting, fishing, sanitary or criminal purposes [36] and may also be used for *D. laxiflora*. Indigenous people use *Dioscorea* species poison in fishing [106]. The main toxic content is dioscorine, an alkaloid present in most *Dioscorea* species [104, 107]. In the domestication process, the physicochemical characteristics of the tuber are the main characteristics that are selected, and the reduction of toxic compounds, for example, becomes a difference between the tubers of wild and domesticated plants [38, 108]. An assessment of the level of toxicity between cultivated plants and the wild conditions of *D. laxiflora* could determine its degree of domestication in this regard.

**Fig. 7:**
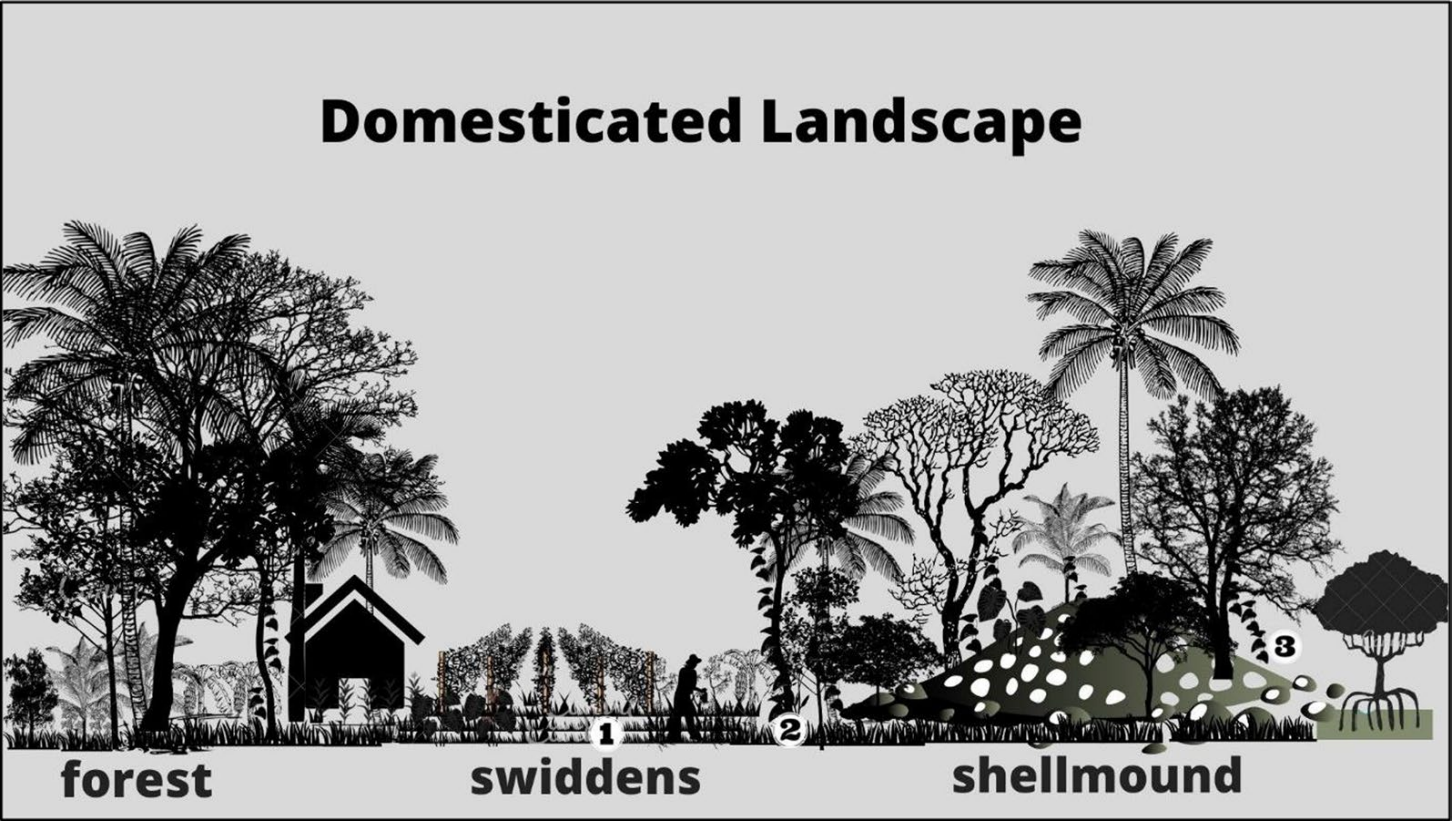
Representation of landscape units where *Dioscorea* species were recorded in the study area: shellmounds and associated vegetation and swiddens. 1: rods with *D. alata, D. bulbifera and D. trifida* in swiddens; 2: *D. cayennensis* inter shellmound and swiddens; 3: *D. chondrocarpa*, *D. dodecaneura, D. laxiflora, D. olfersiana* and *D. scabra* in shellmounds

Just as there are *Dioscorea* species associated with the niche built by shellmound builders, there are domesticated yam species in the region that depend on the niches built by farmers (Fig. 7), cultivated landscape units [109, 110] associated with a domesticated landscape containing species preferred by humans [2]. According to Santos [48], in their swiddens, local farmers reproduce the conditions and means necessary for the vegetative reproduction of the domesticated *Dioscorea* species *D. alata*, *D. bulbifera*, *D. cayennensis* and *D. trifida.* In swiddens, domesticated yams produce tubers from 1.5 kg to 10 kg [44]. These plants are linked to the memory of colonizing families and are considered a cultural asset in the Babitonga Bay region [111]. Although most swiddens are not directly related to the estuary and shellmounds, there is continuity between landscape units. The occurrence of the species *D. cayennensis* associated with three shellmounds is evidence of this continuity. However, the record of only adult and isolated individuals, without evidence of sexual reproduction, is an example of human dependence on reproduction, as also observed by Veasey et al. [44] in secondary forests. The exotic *D. cayennensis* is African and domesticated [42], and its presence in shellmounds in Babitonga Bay is associated with the old swiddens of colonizers that, according to Cunha [80], had dozens of enslaved Africans from the seventeenth century onwards.

In cultivation, yams are wrapped around stakes placed by farmers or around hosts such *as Zea mays* (corn) or around trees in agroforestry systems [48]. *Dioscorea* in the shellmound vegetation and the surrounding forest wrap mainly around hosts, such as small trees, shrubs, herbs and other vines. According to Campanello et al. [112], climbers prefer to use plants with smaller stems first to reach the tree canopy.

Given the presented chronology and the relationship between indigenous peoples, colonizers, and native and exotic species of *Dioscorea* (Figs. 5, 6, 7), it is possible to infer that the native yams that currently occur associated with shellmounds are potentially species that the *Sambaquianos* consumed and whose starch grains were isolated by Wesolowski [30] in Morro do Ouro shellmound. However, given the wide variation in shape and size of starch granules within the same wild species [69, 113] and the small number of granules isolated by Wesolowski [30], it is difficult to determine the exact species and whether it is wild or domesticated. The size and shape of the granules presented in the abovementioned study could belong to the species that we recorded and even to others that we did not find but that could have occurred in the past in the region. Currently, the native species recorded in shellmounds and surrounding forests are in wild conditions. However, this does not rule out human influence in the past. According to Scheel-Ybert [23], considering the macroremains of plants recovered in *sambaquis* and the diversity of food plants in the Restinga, such as fruits, seeds, roots and tubers, it would indicate that the gathering and management was carried out by *Sambaquianos*, but they would be outside an agricultural context. Boyadjian et al. [33] suggest an association between collection and cultivation among the *Sambaquianos* who built the Jabuticabeira II shellmound, south of Santa Catarina, but this hypothesis would still need to be investigated through continued studies and other indicators.

Considering that two studies were carried out with the extraction of microremains of food plants in dental calculi among *Sambaquianos* [30, 32] and that the registered plants have wild relatives, new archaeobotanical research in *sambaquis* is essential to reach specific taxa and, if applicable, confirm horticulture among the *Sambaquianos.*

## Conclusions

Shellmounds and associated forests, and in swiddens, are two contiguous landscape units that have been shaped over thousands of years and are part of a mosaic of cultural legacies.

Native and wild *Dioscorea* occur on shellmounds and exotic and domesticated yams occur in swiddens. They are food and medicinal species of *Dioscorea*.

Considering that the exotic African and Asian species of yam arrived in Brazil only after colonization in the sixteenth century, we can infer that the *Sambaquianos, Jê* and *Guarani* consumed the native species of *Dioscorea* associated with shellmounds, *D. chondrocarpa*, *D. dodecaneura*, *D. laxiflora*, *D. olfersiana* and *D. scabra*, and *D. trifida* associated with swiddens.

The restricted occurrence of *D. dodecaneura*—a semidomesticated species in the Amazon Rainforest—in the *Cubatão* I and *Cubatão* II shellmounds deserves further investigation, especially regarding its distribution in the region.

Greater investment is necessary in research into archaeological remains of plants in *sambaquis*. Of the remains identified thus far, doubts still remain about the taxa and whether they truly are plants that would necessarily need to be cultivated or could simply be gathered and managed in the forests.

There is a lack of studies on native *Dioscorea* species, mainly on their life history and use. Most studies are about taxonomy. Genetic studies conducted within and among *Dioscorea* populations that occur in shellmounds and surrounding vegetation would contribute to the understanding of human manipulations over time. In addition, ethnobotanical studies with yam producers in the region and in communities close to the shellmounds would be complementary and help better understand possible uses of native *Dioscorea* species in wild conditions.

## Data Availability

The data produced by this research may be requested from the author. E-mail addresses provided in affiliations. Full data of the data banks Flora e Funga do Brazil may be accessed at: http://floradobrasil.jbrj.gov.br.

## Abbreviations

BP: Before the present
FOD: Dense Ombrophilous Forest
CUB I-A: Portion in Cubatão I shellmound with forest
CUB I-B: Portion in Cubatão I shellmound covered by grasses and sparse tree vegetation
APG IV: Angiosperm phylogeny classification of flowering plants (2016)
CUB II: Cubatão II shellmound
ECUB II: Cubatão II surrounding area
ILES II: Ilha dos Espinheiros II shellmound
ELES II: Ilha dos Espinheiros II surrounding area
LAS: Lagoa Saguaçu shellmound
ELAS: Lagoa Saguaçu surrounding area
MA II: Morro do Amaral II shellmound
EMA II: Morro do Amaral surrounding area
MO: Morro do Ouro shellmound
RGU: Rua Guaíra shellmound
ERGU: Rua Guaíra surrounding area
CAAE: Certificate of presentation of ethical review
ISE: International Society of Ethnobiology
IPHAN: Institute of National Historical and Artistic Heritage
SAMA: Secretary of Agriculture and Environment of Joinville
UGA: Environmental management unit
FUMDES: State Fund to Support the Maintenance and Development of University Education in Santa Catarina
SC: Santa Catarina state
CNPQ: National Council for Scientific and Technological Development
LAC: Radiocarbon Laboratory of the Universidade Federal Fluminense, Rio de Janeiro, Brazil
